# Effect of counseling on quality of life for children with Moyamoya disease and their caregivers

**DOI:** 10.1186/s12912-026-04756-z

**Published:** 2026-05-20

**Authors:** Sahar Saleh Kamal, Ahmed Abdel-hamid Rihan

**Affiliations:** 1 Pediatric Nursing Department, Faculty of Nursing, Ain Shams University, Cairo, Egypt; 2 Pediatric Medicine Department, Faculty of Medicine, Ain Shams University, Cairo, Egypt

**Keywords:** Moyamoya disease, Quality of Life, Counseling, Pediatric nursing

## Abstract

**Introduction:**

Moyamoya disease (MMD) has a chronic nature which affects physical, cognitive, emotional challenges and relations with others reflecting on the quality of life (QoL) of affected children. Counseling is considered an important supportive intervention that may help children with their caregivers cope with the disease.

**Aim:**

This study aims to evaluate the effect of counseling on quality of life among children with MMD and knowledge of their caregivers. Caregiver outcomes were limited to knowledge assessment.

**Methods:**

A quasi-experimental single-group using paired t-test for pre–post study was conducted at a neurovascular outpatient clinic affiliated with a pediatric hospital in Egypt. A convenience sample of 30 children diagnosed with Moyamoya disease and their caregivers were included. Data were collected prior to counseling and after completion of counseling using a structured questionnaire assessing demographic characteristics and caregiver knowledge, as well as the Pediatric Quality of Life Inventory (PedsQL) for children. PedsQL was transformed to 0–100 scale where higher score means better quality of life.

**Results:**

Before counseling, children demonstrated lower scores PedsQL across health and activities, child feelings, relations with others and school functions. Following the counseling, a statistically significant improvement in PedsQL scores was observed. In addition, caregiver knowledge significantly improved after counseling. A positive correlation was found between children’s PedsQL scores and caregiver knowledge.

**Conclusion:**

Counseling may be associated with improvements in the QoL of children with MMD and enhancement of caregiver knowledge. Further studies using controlled designs and larger samples are recommended to confirm these findings.

## Introduction

Moyamoya disease (MMD) is a vaso-oclusive disorder which affects intracranial vessels with unknown appropriate treatment till now [1]. Moyamoya disease is the main cause of cerebral ischemia presented by hemiplegia, seizures and chronic headache in children [2]. Cerebral ischemia and hemorrhage stroke can be prevented by direct and indirect bypass with revascularization [3]. Vascular anastomosis is difficult in children the brain arteries are very thin. So, indirect anastomosis is more widely used [4].

Moyamoya disease is more prevalent in East Asian countries, particularly Japan, while it remains relatively rare but increasingly recognized in other regions, including the Middle East [5]. Reported incidences of MMD vary between regions: from 0.086/100,000/year in the Western USA to around 1.0/100,000/year in Japan and Korea [28]. Anxiety, depression, low quality of life, poor sleeping are psychological noticeable problems in children with moyamoya disease. However, there are a few studies which reflect the effect of MMD on children [6].

Children have the right to achieve optimal well-being across all aspects of life, including physical, psychological, social, and behavioral domains. Enhancement in these areas collectively contributes to an improved quality of life. Quality of life reflects a multidimensional concept encompassing functional, social, psychological, and physical health factors that influence the overall well-being of children and their caregivers [7]. Children living with chronic and long-term conditions, such as MMD, require continuous support. Such support assists children and their caregivers in coping with stress and strengthens their sense of coherence, which is a key factor in improving the quality of life of children with MMD.

Moyamoya disease is a chronic illness which not affects cerebral vessels only but extends to extra- cranial vessels. It can affect renal vessels causing renovascular hypertension and end-stage renal disease. Therefore, children with moyamoya disease need specialized comprehensive care including counseling to live with chronic life-limiting illness and their families [1].

Pediatric patients with MMD, particularly those younger than five years of age, are at an increased risk of stroke and cognitive impairment due to the involvement of the posterior cerebral circulation in addition to cerebral hypo perfusion [8]. These pathological changes may result in deficits in several cognitive domains, including information processing speed, executive functioning, visuospatial abilities, and memory. Consequently, revascularization procedures are strongly recommended to reduce neurological complications. In addition, educational interventions may contribute positively to cognitive outcomes by enhancing experiential learning, processing speed, and memory, thereby exerting a beneficial effect on overall cognitive function [9].

Various surgical techniques are available for managing MMD, with the most commonly employed procedures being direct, indirect, or combined revascularization approaches [10]. Counseling plays a key role in pediatric nursing care for children with MMD. The educational role of the pediatric nurse is particularly important, as they provide preoperative guidance to children and their caregivers regarding each stage of surgery, postoperative wound care, and pain management. In addition, pediatric nurses deliver direct postoperative care, especially during the first night in the intensive care unit. To minimize the risk of further cerebral ischemic events and seizures, they closely monitor neurological status, arterial blood pressure, and fluid balance [11].

Counseling is the interaction between patients and health care workers providing guidance and information regarding health care and quality of life [12]. Counseling for children with MMD and their caregivers contain carefully listening to their questions. This contributes to accurate assessment of children psychological, social, and cultural issues for MMD. Understanding multidimensional domains of the disease and its effects on whole aspects of life can affect the perception and acceptance of the diagnosis with moyamoya disease [13]. Consequently, it can improve adaptation and quality of life of children with moyamoya disease and their caregivers. Periodic counseling is effective in successful management of children with MMD.

Moyamoya disease affects both physical and mental health aspects in children leading to diminished quality of life. Pediatric patients with aggressive-type MMD are hemodynamically/ neurologically unstable, so they have a history of a neurologic event or significant comorbidities [14]. Therefore, children with MMD may need a referral to psychological or mental health care. Counseling sessions are adjusted to each child patient’s difficulties and their severity, in addition to the child patient’s age. Children with MMD and their caregivers receive individual and group therapy to enhance acceptance and accommodation with the disease [15].

Psychological and emotional support through counseling is vital for children with MMD and their caregivers. Counseling may contribute to improve QOL among pediatric patients through regular emotional assessments, especially if anxiety or depression is high, enhancing coping strategies, improving disease understanding, and strengthening parent–child support [16]. Moreover, strong social support helps reduce stress and improve well-being [17]. Pediatric nurses encourage children to interact with family or support groups to improve mood [18].

Due to the relentless nature of MMD in children, child family especially parents show psychological and emotional stress due to diminished hope in survival. Pediatric nurse are members of health care team providing supportive care through counseling based on family centered approach that can contribute to improve QOL [16]. Pediatric nurse has an essential role in close monitoring, refining outcomes, counseling and follow-up of mental health outcomes among children with MMD [19].

## Methods

### Aim of the study

This study aims to evaluate the effect of counseling intervention on the quality of life of children with Moyamoya disease and the knowledge of their caregivers.

### Hypotheses

The counseling intervention would be associated with improvements in children’s quality of life and enhancement of caregivers’ knowledge.

### Study design

This study used a single group quasi-experimental design with pre and post intervention, which was conducted over a six-month period from February 2025 to July 2025. Data were collected at two time points pre and post counseling. Due to absence of control group, causal inference is limited. Limitations related to internal validity as natural adaptation of children and their caregivers cannot be fully controlled in this design.

### Setting

This study was conducted in the Neurovascular clinic at specialized outpatient clinics affiliated to Pediatric hospital/ Ain Shams University/ Egypt. Children and their caregivers attend for checkup including taking history, clinical neurological examination, review previous tests or request new tests, psychological and educational support. Children and accompanying caregivers were included to support children and help them better understand/ achievement of the study aim.

### Participants

A convenience sample of 30 children diagnosed with MMD, aged 5–12 years and their caregivers. The studied children attended to the clinic received pre and post care. Children with MMD exposed to surgical procedures or not were all included in the study. It was considered only to guide the tailoring of counseling content according to the child’s clinical condition and it may have influenced the outcomes representing a potential confounding factor.

Inclusion criteria:

- Confirmed diagnosis of Moyamoya disease.
- Attendance at outpatient follow-up clinic.
- Both pre and post-operative children.

Exclusion criteria:

- Children with other neurodevelopmental diagnosis.

### Sample size

The study included all eligible children diagnosed with Moyamoya disease who attended the neurovascular outpatient clinic during the data collection period (February 2025 to July 2025). A total of 30 children who met the inclusion criteria and agreed to participate were recruited. A convenience sampling technique was used, and all eligible cases during the study period were included (total enumeration).

### Tools for data collection

Tool I was a pre-designed questionnaire consisting of three parts; the first part collected demographic characteristics of children with MMD using six items as age, gender, level of education, while the second part collected demographic characteristics of caregivers using 3 items as age and educational level. The third part is the assessment knowledge tool consisted of 6 closed ended items as what is MMD and causes of MMD to assess knowledge of caregivers about MMD pre and post counseling. Each item was scored on a 3 point scale (0 = incorrect answer, 1 = partially correct and 2 = correct). Total score ranged from 0 to 12 score with higher score indicate better knowledge.

Knowledge assessment tool was developed by the researchers after reviewing the relevant literature [20]. Content validity was established by a panel of experts in pediatric nursing and neurology, who assessed the tool for clarity, relevance, and comprehensiveness. Modifications were made accordingly. The tool was translated into Arabic using the forward–backward translation method. It was translated into Arabic by a bilingual expert, revised and any discrepancies were resolved to ensure accuracy and consistency.

Tool II: Pediatric Quality of Life Inventory (PedsQL) ages 5–7 and 8–12 versions Generic Core Scales, adopted from Varni & Limbers [21] was used in its age-appropriate forms for children aged 5–12 years. For younger children (5–7 years), the PedsQL Scale was administered through face-to-face structured interviews by the researchers. Children aged 8–12 years completed the self-report form independently, with assistance provided when necessary. To overcome literacy limitations, items were read aloud and explained in a standardized manner without leading the participants.

Pediatric Quality of Life Inventory (PedsQL) Generic Core Scales was used to assess health-related quality of life among children with MMD pre, post counseling. PedsQL 23 items were reverse-scored and linearly transformed to a 0–100 scale, with higher scores indicating better quality of life, according to the developer’s guideline. Each item is scored on a 5-point Likert scale (0–4), then reverse-scored and transformed to a 0–100 scale: 0 = 100–1 = 75 − 2 = 50 − 3 = 25 − 4 = 0 where higher scores indicate better quality of life.

The PedsQL includes four domains: Physical, emotional, social and school functioning. Physical functioning includes health and activity which consisted of 8 items as the child has a problem in participating in a game or exercise, taking a bath or shower. Emotional functioning is the child feelings included 5 items assessing problems with feelings such as fear and anger. Social functioning included 5 items assessing problems with interactions with other children, such as getting along and keeping up during play. School functioning included 5 items assessing problems with attention in class and keeping up with schoolwork.

**Table:** 

	No of items	Cronbach’s alpha
Physical Health	8	0.621
Mental Health	5	0.696
Social Functioning	5	0.735
School Functioning	5	0.682
Total QOL	23	0.723
kn	6	0.763

The Cronbach’s alpha values indicated acceptable to good internal consistency across all domains, confirming the reliability of the study instruments in the current sample.

### Intervention

Description of the Counseling Intervention (TIDieR Checklist).

The counseling intervention is introduced in accordance with the TIDieR checklist to ensure clarity and reproducibility.

#### Brief name

A structured counseling intervention provided for children with moyamoya disease (MMD) containing knowledge for their caregivers about MMD and its home management.

#### Why (Rationale)

Counseling intervention was designed to contribute to improving quality of life among children with MMD. In addition, caregivers/ parents of children included knowledge based intervention that can improve knowledge regarding disease nature, management, warning signs, treatment adherence, and home care practices of caregivers toward their children with MMD.

#### Materials

The intervention utilized:

The study team developed an interview guide included a short oral presentation about the MMD counseling that corresponded with a booklet distributed on the study group both children and their caregivers both mothers or fathers and asked them to review before the interview. Counseling intervention was maintained by using a standardized protocol that clearly outlined the content, duration, and structure of each session.

#### Procedures

The intervention was delivered in three sequential stages:

##### Pre-counseling stage (Baseline assessment)

During baseline assessment sessions in the first month, baseline demographic and clinical data were gathered using a pre-designed questionnaire. In addition, knowledge of the caregivers was assessed about MMD and its medical management (Tool 1) however; caregiver quality of life or psychological outcomes were not directly measured. The quality of life of children with MMD was assessed through Pediatric Quality of Life Inventory (PedsQL) containing physical, emotional, social, and school functioning (Tool II). Pediatric Quality of Life Inventory evaluated physical aspects (as problems with walking, running) psychological functioning (problems with feeling of each child as feel afraid, sad, angry). Additionally social functioning (problems with getting along with other kids, other children annoying him) and school functioning (as it is hard to interact with teachers and have absent from school).

##### Counseling stage

The sessions included knowledge about MMD as definition, causes, complications and better home management. Caregivers received education on disease management, long-term care, and daily living modifications to reinforce learning and support home implementation. In addition, counseling sessions contain lifestyle guidance, and emotional support, social and educational support. The counseling sessions were provided for children and their caregivers depending on the child age especially preschool age children where caregivers / parents were included essentially in the counseling. Sessions incorporated simplified language, visual aids, and interactive activities appropriate to children’s age.

##### Post-counseling stage

Knowledge of caregivers about MMD was re- assessed after completion of counseling intervention (Tool I). In addition, quality of life of children was re- assessed after counseling using the (PedsQL) Generic Core Scales (Tool 2). Post-intervention data were collected one month after completion of the counseling program.

#### Who provided

The counseling sessions were delivered by the research team (pediatric nurse professional and the physician). All sessions were conducted by the same researchers to ensure consistency.

#### How (Mode of Delivery)

Sessions of counseling delivered face-to-face to children and their caregivers. Caregivers were actively involved, especially for younger children (preschool age).

#### Where

The intervention was conducted at the neurovascular clinic at specialized outpatient clinics affiliated to Pediatric Hospital, Ain Shams University, Egypt.

#### When and how much

The study was conducted over six months. The counseling intervention was delivered over the subsequent five months through twice weekly sessions (approximately 48 sessions in total). Each session lasted approximately 30–45 min. Time interval between pre- and post-assessment: approximately 6 months.

#### Tailoring

Minor tailoring was applied in counseling intervention based on the child’s clinical status. Children who had undergone surgery received additional postoperative-related counseling as activity precautions and recognition of postoperative complications. Non-operated children received more emphasis on symptom recognition, disease progression, and preparation for possible surgical management. This tailoring was intended to enhance the relevance of the intervention rather than to create distinct comparison groups.

#### Modifications

More details were added and clarified. No major modifications to the intervention protocol were reported during the study.

#### How well (Planned – Fidelity Assessment)

Fidelity was ensured through use of a standardized protocol outlining session content and structure, delivery of all sessions by the same researcher and consistent session duration and frequency.

#### How well (Actual)

All participants (*n* = 30 children and their caregivers) received the intervention. Caregivers attended sessions to reinforce knowledge and support home implementation. No long-term follow-up beyond one month post-intervention was conducted.

### Ethical considerations

The study was carried out in compliance with established ethical standards for research. Ethical approval was granted by the Scientific Research Ethical Committee of the Faculty of Nursing, Ain Shams University (Approval No. 25.10.961) prior to the start of data collection. Informed written consent was obtained from the children caregivers after they received a clear and age-appropriate explanation of the study objectives and expected outcomes to ensure full understanding. Participation in the study was entirely voluntary, and participants were informed of their right to withdraw at any stage without any obligation or consequences. They were also assured that participation in the study would not cause any harm to them or to children. Confidentiality and anonymity.

### Statistical analysis

Data were analyzed using SPSS software (version 26). Descriptive statistics were presented as mean ± standard deviation (SD) for continuous variables and frequency with percentage for categorical variables. Paired t-test was used to compare pre- and post-intervention mean scores within the same group. Pearson correlation coefficient was applied to assess the relationship between total quality of life and total knowledge scores before and after the intervention.

A p-value of < 0.05 was considered statistically significant.

## Results

This quasi-experimental study included 30 children with moyamoya disease; seven were male, with a mean age of 8.15 ± 3.07 years. Over two-thirds of the children studied (66.7%) diagnosed with moyamoya disease from less than one year and did not have surgery before. Clinically, 26.7% had undergone previous surgery. The majority of them (93.3%) not receive psychological / social support (Table 1).

**Table 1:** Frequency distribution of demographic data for the 30 children studied (n = 30)

Items	Frequency (n)= 30	
	N	%
Chronological age (in years)		
M ± SD	8.15±3.07	
Gender		
Female	23	76.7
Male	7	23.3
Level of Education		
Preschool	7	23.3
Primary school	14	46.7
Preparatory school	9	30.0
Surgery done before		
Yes	8	26.7
No	22	73.3
Duration of diagnosis of Moyamoya disease:		
Less than 1 year	20	66.7
More than 1 year	10	33.3
The child receive psychological / social support"		
Yes	2	6.7
No	28	93.3

Table 2 shows that the caregivers’ mean age was 29.12 ± 5.44 years. Although 60% of them have moderate education, 53.3% of them are not working that the majority of them were mothers.

**Table 2:** Frequency distribution of demographic data among the caregivers of the children studied (*n* = 30)

Items	Frequency *n* = 30	
	*N*	%
Chronological age (in years):		
Mean ± SD	29.12 ± 5.44	
Educational level:		
Illiterate Moderate educated Highly educated	4 18 8	13.3 60.0 26.7
Employment status:		
Work Not work	14 16	46.7 53.3

Table 3 shows an increase in the mean total PedsQL score in post (36.47 ± 3.16) than pre counseling (29.63 ± 4.75). There is a significant difference (t = 13.462, *p* < 0.001) indicating that counseling can contribute in improving quality of life of children with moyamoya disease. All domains demonstrated large effect sizes (Cohen’s d > 0.8), with the highest values observed in physical health and total quality of life, indicating that counseling can contribute in improving quality of life of children.

**Table 3:** Total quality of life (PedsQL) of children with Moyamoya disease

Domains of PedsQL	Pre (*N* = 30)	Post (*N* = 30)	Paired t-test		Cohen’s d
			t	*P*-value	
Physical Health	29.17 ± 6.76	37.29 ± 5.62	13.355	< 0.001*	2.44
Mental Health	31.17 ± 6.78	37.00 ± 5.35	6.727	< 0.001*	1.23
Social Functioning	30.50 ± 9.41	37.00 ± 6.51	7.779	< 0.001*	1.42
School Functioning	27.17 ± 10.23	34.33 ± 5.98	6.420	< 0.001*	1.17
Total Quality of Life	29.63 ± 4.75	36.47 ± 3.16	13.462	< 0.001*	2.46

Table 4 shows an increase in the mean total knowledge score from pre (4.07 ± 0.64) to post counseling (6.97 ± 1.19). There is a significant difference (t = 12.517, *p* < 0.001) indicating that counseling can improve knowledge of caregivers about care of their children with moyamoya disease. An effect size was observed (Cohen’s d = 2.29)

**Table 4:** Total Knowledge of caregivers about Moyamoya disease

Total Knowledge Score	Pre (*N* = 30)	Post (*N* = 30)	Paired t-test		Cohen’s d
			t	P-value	
	4.07 ± 0.64	6.97 ± 1.19	12.517	< 0.001*	2.29

Table 5 shows a statistically significant positive correlation between total quality of life and total knowledge scores, a weak to moderate positive correlation was observed (*r* = 0.372, *p* = 0.043). After the intervention, this relationship became stronger, showing a moderate to strong positive correlation (*r* = 0.610, *p* < 0.001).

**Table 5:** Correlation between total knowledge of caregivers about Moyamoya disease and total quality of life (PedsQL) of their children

Total QOL	Total Knowledge	
	*r*	*P*-value
Pre	0.372	0.043*
Post	0.610	< 0.001*

## Discussion

This study aimed to assess the effect of counseling on the quality of life of children with moyamoya disease and knowledge of their caregivers. The findings of this study suggest that counseling was associated with improvements in quality of life aspects of physical, emotional, social and school functioning among children with Moyamoya disease and knowledge of their caregivers. These findings may be explained by improved disease understanding and coping strategies provided during counseling. The improvement in caregiver knowledge highlights the importance of family-centered care in managing chronic pediatric conditions. However, findings should be interpreted with caution due to absence of control group and small sample size.

Surgical status was not analyzed as a factor influencing outcomes, as the intervention was designed to be individualized rather than comparative. However, differences between preoperative and postoperative children may have influenced responses to counseling and should be explored in future studies.

The results revealed that children with moyamoya disease had problems in physical function as bathing/showering ability, walking, sports participation, running ability, lifting heavy objects, pain, and low energy pre-counseling which decreased in post –counseling. There is a statistical significant difference in children health and activities pre / post –counseling. Children had problems in emotional health as feeling sad, worry about the future, scared of medical team members and trouble sleeping before counseling. There is a statistical significant difference in children feelings pre/ post-counseling indicating that counseling may have contribution to relieve children problems with their feelings. However, given the quasi-experimental design and absence of a control group which is recommended in future studies, these findings should be interpreted with caution, and causality cannot be firmly established.

Regarding relation with others, children had problems as trouble getting along with other children, other children avoid him, other children tease him; struggle to keep up when playing with other kids. In addition, children had problems in school function as it is hard to keep up with other children, children had poor school achievement, forgot things, and missing school to see the doctor or go to the hospital pre-counseling. After providing sessions of counseling, there is an increase in the mean total PedsQL score in post than pre counseling. There is a significant difference indicating that counseling can contribute to improve quality of life of children with moyamoya disease. This may be due to lack of support provided for those children that they not need only medical intervention but also they need knowledge about moyamoya disease and their caregivers, physical and emotional support besides receiving medical intervention. Additionally, potential confounding factors such as surgical status, duration since diagnosis, and access to external psychological support may have influenced the observed outcomes. The study is also limited by its small sample size and single-center setting, which may restrict the generalizability of the findings, Therefore, the results should be considered preliminary.

The study conducted by Andere et al. [22], supports the current study results who reported that children with moyamoya disease experienced headache, anxiety and pain attack which contributed to functional neurologic disorder. Psychological and functional neurologic disorders can be similar with stroke symptoms in children with moyamoya arteriopathy, leading to misdiagnosis and eventually poor quality of life.

Also, Rajamani et al. [23], who detected that impaired physical aspects as poor eating, growth and developmental deterioration and involuntary movements may result from decreased brain blood supply in children with Moyamoya disease.

Oakley et al. [24], at his study revealed that the most disabled and severe neuropsychiatric symptoms may present in children with moyamoya disease are psychosis resembling schizophrenia. Follow up the condition of children with MMD is essential for early recognition of MMD and treatment can significantly be associated with improvement quality of life in children and their caregivers.

Similarly, the findings of the present study are consistent with those reported by Ganesan et al. [1], who emphasized the importance of continuous follow-up and ongoing management for children with MMD. Their work highlights the need for comprehensive supportive care, including counseling, blood pressure regulation, and nutritional management. Such care should be delivered through a multidisciplinary healthcare team and involve family-centered approaches within counseling sessions. Implementing this multidimensional management strategy may play a significant role in enhancing the quality of life of children with MMD.

Moreover, Dlamini et al. [25], showed that children with moyamoya disease have arterial ischemic which is presented as headache and cognitive impairment, overall decrease in blood supply in circulation, loss of movements, seizures besides fatigue and crying.

In the same way, regarding the psychological status of children with moyamoya disease, Oh et al. [26], was consistent with the present results revealed that children with MMD had negative feelings of tension, worried and anxiety accompanied with the physical symptoms.

Similarly, Yang et al. [18], who conducted that children patients with MMD had poor psychological condition and stress, can be managed through identifying the predictors to improve quality of life. Although these results are applied on adult patients, it is recommended to be applied also on children to improve quality of life.

The study also showed that children had a poor quality of life both before and after counseling regarding their academic achievement. Before counseling, children with moyamoya disease had significantly lower scores for school-related performance, particularly in areas like difficulty paying attention in class and forgetting things, compared to after counseling. A significant difference was found between pre- and post-counseling concerning child relationships with others and school functioning. These findings align with those of Ahmad et al. [6], who reported that anxiety, worry, and memory difficulties are the most common symptoms in children with moyamoya disease, all of which contribute to a diminished quality of life.

Children with MMD had poor quality of life and this keeps them in need to psychological, mental health care and physical health care which can be only provided through counseling. This result is in accordance with results reported by Alexander [27] who revealed that, children with moyamoya disease are in need of psychological and mental support rather than physical support.

As regards to providing counseling for children with moyamoya disease and knowledge for their caregivers, the results of the current study revealed an increase in the mean total knowledge score from pre to post counseling. There is a significant difference indicating that counseling can improve knowledge of caregivers about care of their children with moyamoya disease. During counseling sessions, health care providers especially pediatric nurse assess family history, child patient needs and their caregivers through an interactive process. Pediatric nurse provide information about the child health status and help them to make the best decision according to their needs and expectations to achieve the highest possible level of quality of life. For children with MMD and their caregivers, pediatric nurse provide instructions in counseling sessions. Instructions include food regimen, sleep pattern, emotional and relaxation technique, school achievement and peer relationship, social support and referral.

These results align with the findings of Ganesan et al. [1], who emphasized the need for regular periodic counseling sessions for children with moyamoya disease, as these sessions can significantly contribute to better disease management. Ganesan et al. also highlighted the importance of collaboration among various healthcare professionals, including physicians, specialized nurses, surgeons, psychologists, and social workers, to contribute to improve the quality of life in multiple aspects—physical, psychological, and social—for both the children and their caregivers.

These results are also supported by the study conducted by Ahmad et al. [6], who found that children with moyamoya disease (MMD) often lack sufficient awareness about their healthcare, including the management of MMD and its consequences, which hinders optimal health outcomes. As a result, these children require counseling that may improve both their quality of life and healthcare outcomes. There is an urgent need for widespread screening in children with MMD. A better understanding of their patient-reported needs and outcomes, along with the provision of comprehensive support in physical, psychological, mental, and social care, is essential for enhancing their overall well-being.

Moreover, counseling is recognized as essential in the French clinical practice guidelines for Moyamoya angiopathy, as outlined by D. Herve´ et al. [28]. The guidelines emphasize that children with MMD and their families require psychological support. Such support can help mitigate the psychological consequences of the disease and address personal, professional, and family challenges that may arise.

In addition, systematic review collected by Cobham et al. [29] about anxiety in children and adolescents with chronic diseases includes multiple studies which examined the incidence of anxiety and psychological disorders. It paid attention to the importance of improving knowledge about disease, health care, psychological support and family centered care due to raising quality of life for those children with chronic diseases as MMD. It can be achieved successfully during the counseling sessions.

### Implications for practice

The results of this study emphasize the importance of integrating counseling into pediatric care to enhance healthcare outcomes; may improve the quality of life for children with moyamoya disease and knowledge of their caregivers. Counseling can help improve health, daily activities, psychological well-being, peer interactions, and school performance, leading to better overall quality of life. Each child with MMD may have different needs, and a thorough health assessment by the pediatric healthcare team is essential. This includes evaluating intracranial blood supply, blood pressure, cognitive issues, movement problems, headaches, learning difficulties, and social or emotional concerns. Addressing these requires coordinated care from the entire medical team, with counseling playing a central role. Counseling incorporates both psychological and physical therapy through collaboration of multidisciplinary team of care providing. It can contribute to alleviate the psychological impact of MMD on the child and their caregivers. School support can help manage learning challenges and improve academic performance. Future research should focus on the effectiveness of child-centered care and integrated team efforts in supporting children with moyamoya disease. Also, future randomized controlled studies to confirm the findings are recommended.

### Limitations

Despite the valuable findings of this study, some limitations should be acknowledged. The study sample was limited due to time constraints, which may affect the generalizability of the findings. Future studies should consider a larger sample size over a longer period to improve the reliability of the results. Additionally, counseling needed long term evaluation to assess long term health care outcomes. Future research should incorporate extended follow-up periods to better evaluate the long-term effectiveness of counseling interventions on children with moyamoya disease and their caregivers. As well as, the use of one-group quasi-experimental design without a control group may limit the ability to establish causal relationships. Future studies using randomized controlled designs are recommended to confirm causal relationships. Also, caregiver-related outcomes such as quality of life, burden, or psychological distress were not evaluated, despite caregiver involvement in the intervention. The child’s clinical condition (have surgery or not), it may have influenced the outcomes representing a potential confounding factor and limitation. Due to the small number of surgically treated children, subgroup analysis was not feasible. Therefore, surgical history should be considered as a potential confounding factor when interpreting the study finding.

## Conclusion

In conclusion, counseling was associated with increased knowledge of children caregivers about moyamoya disease. There were significant differences between pre- and post-counseling scores regarding physical health, emotional health, child relationships with others and school functioning and total QOL among children with moyamoya disease. These findings suggest that counseling may contribute to improvements in the quality of life of children with moyamoya disease and knowledge of their caregivers.

## Data Availability

All data available at the manuscript.

## Abbreviations

MMD: Moyamoya disease
QoL: Quality of Life
PQLIS: Pediatric Quality of Life Inventory Scale
SPSS: Statistical Package for Social Sciences
TIA: Transient Ischemic Attacks
